# The Role of *Twist2* in Myoblast Proliferation, Fusion, and Its Impact on Muscle Structure During the Growth of Chinese Perch (*Siniperca chuatsi*)

**DOI:** 10.3390/ani15081177

**Published:** 2025-04-20

**Authors:** Yangyang Meng, Wei Zeng, Xin Zhu, Lingsheng Bao, Yaxiong Pan, Honghui Li, Jianshe Zhang, Lusha Liu, Zexia Gao, Zhenyu Du, Wuying Chu

**Affiliations:** 1 College of Biological and Chemical Engineering, Changsha University, Changsha 410022, China; mengyangyang163938@163.com (Y.M.); zengwei_2014@163.com (W.Z.); baolingsheng2003@126.com (L.B.); biopyx@163.com (Y.P.); lee19890925@163.com (H.L.); jzhang@ccsu.edu.cn (J.Z.); 2 Key Lab of Agricultural Animal Genetics, Breeding and Reproduction of Ministry of Education, Key Lab of Freshwater Animal Breeding, Ministry of Agriculture, College of Fisheries, Huazhong Agricultural University, Wuhan 430070, China; liuls@mail.hzau.edu.cn (L.L.); gaozx@mail.hzau.edu.cn (Z.G.); 3 Laboratory of Aquaculture Nutrition and Environmental Health, School of Life Sciences, East China Normal University, Shanghai 200241, China; zydu@bio.ecnu.edu.cn

**Keywords:** *Twisit2*, skeletal muscle, myoblast, *Siniperca chuatsi*

## Abstract

*Twist2* plays a pivotal regulatory role in the growth of skeletal muscle across various organisms. Nonetheless, the specific mechanism by which *Twist2* governs skeletal muscle function in fish, particularly in the economically significant Chinese perch (*Siniperca chuatsi*), remains unclear. Within the muscle injury model in Chinese perch, we observed that *Twist2* expression was upregulated during the repair phase of fast muscle tissue, exhibiting an expression pattern analogous to that of *Pax7*. Following the knockdown of *Twist2* using *Twist2*-specific in vivo-siRNA in fast muscle tissues, the expression of myogenic regulatory factors (*MRFs*) *and Myomaker* was significantly reduced in the *Twist2*-siRNA-treated group compared with the control group, whereas no significant differences were observed for *Pax3* and *Pax7*. Furthermore, the diameter of myofibers and the number of nuclei in single myofibers were reduced, and concurrently, the number of BrdU-positive cells (proliferating cells) was significantly reduced in the *Twist2*-siRNA-treated group. Taken together, this study demonstrates that Twist2 promotes myoblast proliferation and fusion, thereby regulating fast muscle growth in juvenile Chinese perch. These findings provide a clear direction for further exploration of molecular mechanisms underlying skeletal muscle growth in economic fish species.

## Simple Summary

*Twist2* is crucial for the growth of skeletal muscle in organisms. However, the role of the *Twist2* gene in the growth of skeletal muscle of economically important fish during the post-embryonic stage remains unclear. This study showed that in the muscle injury model of Chinese perch, *Twist2* expression increased during the rapid muscle repair phase. After in vivo knockdown of *Twist2* in fast muscle, the expression of myogenic regulatory factors (MRFs) and *Myomaker* was significantly reduced, while there was no difference in the expression of *Pax3* and *Pax7*. In addition, the diameter of muscle fibers, the number of nuclei in single muscle fibers, and the number of BrdU-labeled myoblasts were all significantly reduced. These findings indicate that *Twist2* can promote the proliferation and fusion of myoblasts, thereby facilitating the growth of fast muscle in juvenile Chinese perch.

## 1. Introduction

Skeletal muscle, as the main structural tissue, is not only involved in the coordination of movement but also plays an important role in regulating its own metabolism and maintaining physiological health [1,2,3]. Regarding the composition of the fish body, skeletal muscle constitutes 40–60% of the fish’s body weight [4]. Based on anatomical and physiological characteristics, skeletal muscle can be classified into glycolytic fast muscle and oxidative slow muscle [5]. Fast muscle represents the primary part of the skeletal muscle in fish. It is situated in the deep layer of the trunk and constitutes approximately 95% of the total muscle mass in fish [6]. In contrast, slow muscle is distributed in a wedge-shaped, superficial strip along the lateral line, parallel to the longitudinal axis of the trunk [7]. The growth efficiency of fish skeletal muscle and its accumulation of muscle content have a significant impact on the economic efficiency of aquaculture [8,9]. Therefore, an in-depth investigation of the molecular regulatory mechanisms of fish fast muscle growth is of crucial significance for the development of fish farming. In vertebrates, muscle growth patterns are categorized into deterministic and non-deterministic growth, whereas in fish, most of the commercially important production fish exhibit non-deterministic growth, except for zebrafish, which exhibits deterministic growth that is more similar to mammalian growth [10]. Non-deterministic growth in fish is typified by the capacity for perpetual muscle growth across the lifespan, which is manifest as an increase in the number of myofibers (hyperplasia) and enlargement of the cross-sectional area of each single myofiber (hypertrophy), occurring both pre- and post-hatching stages [11]. At present, there is limited understanding of post-hatching growth of fast muscle in fish, especially in species with non-deterministic growth patterns such as the Chinese perch. As one of China’s major economic fish species, the Chinese perch is commonly found in freshwater rivers and lakes, and it is renowned for its delicious flesh. Similar to other non-determinate growth fish species, the Chinese perch, being a fast-growing freshwater fish, undergoes throughout its life cycle muscle fiber hyperplasia and hypertrophy [12]. Therefore, investigating the regulatory mechanisms of hyperplasia and hypertrophy in fast muscle fibers of the Chinese perch can not only reveal the molecular basis of its growth patterns but also fully exploit its growth potential to enhance fishery production.

The complex process of myofiber hyperplasia and hypertrophy involves the proliferation and fusion of myoblasts, a process that is regulated by multiple muscle-specific transcription factors and protein families [13,14]. Proliferation is dependent on quiescent satellite cells that become myoblasts through activation and undergo self-renewal or differentiation, whereas *Pax7* is a satellite cell marker gene that is essential for the establishment of the satellite cell pool [15]. Myofiber hypertrophy occurs through the fusion of mononuclear myoblasts to form new multinucleated myofibers, and *Myomaker* is one of the known muscle-specific proteins that are absolutely essential for the fusion of embryonic and adult myoblasts [16]. In addition to this, myogenic regulatory factors (MRFs), *Myf5*, *MyoD*, *MyoG,* and *MRF4*, have important functional roles in myogenic lineage determination and muscle differentiation, with distinct temporal and spatial features of activation of *MRFs*, while myoblast differentiation is activated by the regulatory sequence of *MRFs* [17,18]. However, the molecular mechanisms by which myogenesis regulators regulate muscle growth in fish during the post-hatching stage have not been identified.

*Twist* was first identified in Drosophila in 1987 and has been shown to have an important role in the establishment of dorsoventral patterns in the early embryo [19]. In vertebrates, the *Twist* family consists of two members, *Twist1* and *Twist2* [20], and because *Twist2*-expressing myogenic progenitor cells are a newly discovered fiber-type-specific stem cell, *Twist2* has attracted more attention in the direction of myogenic research than *Twist1* [21]. It was reported that *Twist2* promotes the formation of type IIb/x fiber types in fast muscles during muscle regeneration and that Twist2^+^ cells can autonomously initiate myogenesis, fusing with themselves and muscle satellite cells to regenerate and repair damaged tissues. Previously, it was generally believed that muscle regeneration after injury was mainly dependent on Pax^+^-labeled satellite cells, and the findings of *Twist2* fill the gap that there is a group of myogenic progenitor cells specialized in generating specific fiber types and are involved in regulating the regeneration process. However, *Twist2* remains a gap in research on skeletal muscle growth in fish. The specific expression pattern of *Twist2* in fish during the muscle regeneration process, its function in the regulation of myogenesis-related factors, and its overall impact on muscle growth remain to be elucidated.

To explore the functional role of *Twist2* in the myogenesis of Chinese perch (*Siniperca chuatsi*), here, we revealed the expression profile of *Twist2* during muscle regeneration in Chinese perch juveniles. After knocking down *Twist2* using *Twist2*-specific in vivo siRNA, we analyzed the expression changes of genes related to muscle growth, observed the alterations in histological characteristics of muscles, and detected the proliferation and fusion of myoblasts so as to clarify the regulatory mechanism of *Twist2* on the fast muscle growth of Chinese perch. This study can enrich the basic theory of skeletal muscle growth in fish during the post-hatching stage, thus providing a scientific basis for the breeding of Chinese perch.

## 2. Materials and Methods

### 2.1. Experimental Animals

The Chinese perch (2-month-old, body weight 6 ± 0.5 g) used for the muscle injury model and siRNA interference experiments were obtained from Hunan Fisheries Science Institute (Changsha, China), which all originated from the same parents and belonged to the same breeding lot. Chinese perch were maintained in a recirculating aquarium system for scientific collective rearing (water temperature of 25 ± 2 °C, dissolved oxygen level of 7.9 ± 0.3 mg/L, pH range of 7.5 ± 0.3) and fed equal amounts of *Cirrhinus mrigala* larvae twice a day (9:00 a.m. and 6:00 p.m.). This study was approved by the Animal Care and Use Committee of Changsha University (approval number 2024043), and the animal experimentation procedures were in accordance with the ARRIVE guidelines.

### 2.2. Design and Preparation of siRNAs

Obtaining the complete CDS sequence of Chinese perch *Twist2* from the Chinese perch genome database (http://genomes.igb-berlin.de/Siniperca/ (accessed on 21 May 2024)). The siRNA (*Twist2*-specific in vivo siRNA) targeting *Twist2* modified with cholesterol (sense 5′-GCUGGACAGACAGCAGAAATT-3′, antisense 5′-UUUCUGCUGUCUGUCCAGCTT-3′) and nonsense siRNA modified with cholesterol (referenced from previous studies) were used as controls [22]. Individual cholesterol molecules were linked to the 3′ end of the passenger strand. *Twist2*-siRNAs were designed and synthesized by Shanghai GenePharma Co., Ltd (Shanghai, China).

### 2.3. Muscle Injury Model Construction, Twist2-siRNA In Vivo Inhibition Assay, and Tissue Sampling

Chinese perch used for muscle injury were injected with Cardiotoxin Analog (CTX) (MedChemExpress, Shanghai, China) at the first segment of the dorsal fast muscle at a concentration of 10 μM, with a volume of 20 μL [23]. Sampling was conducted at four specific time points: 0-, 1-, 3-, and 7 days post-injection, and anesthesia was administered to Chinese perch using tricaine mesylate (MS-222, 0.15 mg/mL). Six Chinese perch were collected on ice at each time point, and the sampling site was the first dorsal fast muscle segment. Three Chinese perch from the control group and the experimental group were randomly selected for histological analysis and gene expression profiling, respectively.

In the *Twist2*-siRNA study, Chinese perch were allocated completely randomly to control and *Twist2*-siRNA interference groups, with each group consisting of six fish. The interference dose of *Twist2*-specific in vivo siRNA was determined according to a previous experimental report [22]. The *Twist2*-specific in vivo siRNA was injected once, and the same dose of nonsensical siRNA was injected into the control group. Furthermore, 7 days later, 10 mg/kg BrdU was injected into the dorsal muscle of Chinese perch at 12 h before sampling. Anesthesia was administered using tricaine mesylate (MS-222, 0.15 mg/mL), and then the Chinese perch dorsal skin was gently separated from the underlying skin at the second dorsal muscle segment using a scalpel and forceps. Regarding the processing and preservation of tissue samples, all the fast muscle samples were divided into two parts. One part was stored at −80 °C for RNA extraction, and the other part was fixed with 4% paraformaldehyde at 4 °C overnight for tissue sectioning and immunofluorescence analysis.

### 2.4. cDNA Synthesis and Quantitative Real-Time PCR Analysis

Total RNA from all fast muscle samples was extracted utilizing the RNAiso Plus (9109, Takara, Beijing, China), and the extracted RNA’s concentration and integrity were evaluated by ultramicro spectrophotometer (Thermo NanoDrop One, Thermo Fisher, Waltham, MA, USA), and the integrity of the RNA was detected by 1.5% agarose gel electrophoresis. Equal amounts of RNA were reverse transcribed using MonScript ^TM^ RTIII Super Mix with dsDNase (Monad, Beijing, China). The qRT-PCR was detected using SYBR Premix Ex TaqTM (TaKaRa, Beijing, China). The qPCR method was applied as previously reported [24]. The relative expression level of the target mRNA was determined by *R* = 2^−∆∆Ct^ calculation [25]. The qRT-PCR primers were designed by the Primer-BLAST tool on the NCBI webpage, and the primers were synthesized by Beijing Tsingke Biotechnology Co., Ltd. (Beijing, China). The sequences are shown in Table 1.

### 2.5. Isolation of Single Muscle Fibers and DAPI Staining

In each of the control and experimental groups, three Chinese perch were completely randomly selected, and 20 single muscle fibers at the dorsal fast muscle were taken from each fish. The isolated single muscle fibers were selected under a stereomicroscope (Stemi508, Zeiss, Jena, Germany) and placed on slides coated with poly-L-lysine. Staining was performed using DAPI (BL105A, Biosharp, Hefei, China) at a concentration of 1 µg/mL, and after 10 min of staining, single muscle fibers on each slide were triple-washed for 5 min each with PBST. Photographs were taken with a fluorescence microscope (DMI3000B, Leica, Wetzlar, Germany).

### 2.6. Histological Section and Immunofluorescence Analysis

Fast muscle samples were dehydrated with gradient ethanol solution and embedded in paraffin, sliced into 6 µm size using a Leica HistoCore BIOCUT slicer (Leica Biosystems, Milton Keynes, UK), placed on poly-L-lysine, dewaxed, and then stained using a Hematoxylin and Eosin Staining Kit (C0105S, Beyotime, Shanghai, China) in accordance with the manufacturer’s guidelines, with a special note of 10 min for hematoxylin staining and 30 s for eosin staining. For immunofluorescence detection, paraffin sections were deparaffinized and antigenically repaired with Antigen Repair Solution (Sangon Biotech, Shanghai, China). The muscle sections, along with the single muscle fibers, were then subjected to immunofluorescence labeling to identify proliferating cells within the muscle tissue, following a previously described protocol [26]. Tissue sections and single muscle fibers were incubated overnight with anti-BrdU antibodies: Servicebio (GB12051, Wuhan, China) for tissue sections and Abcam (ab152095, Cambridge, UK) for single muscle fibers. All samples were then incubated with fluorescence-conjugated secondary antibodies: Servicebio (GB21301, Wuhan, China) for tissue sections and Abcam (ab150077, Cambridge, UK) for single muscle fibers. Finally, nuclei in all samples were co-stained with DAPI (BL105A, Biosharp, Hefei, China). The observation of tissue sections was conducted using an inverted fluorescence microscope (DMI3000B, Leica, Germany). The acquired images were processed and analyzed with ImageJ 1.50i software.

### 2.7. Statistical Analysis

The data were statistically analyzed by IBM SPSS Statistics version 26. Unless otherwise specified, all data are presented as mean ± SEM. The significance of the difference between two groups was analyzed by an independent-sample t-test, and significant differences between the two groups were defined as *p* < 0.05.

### 2.8. Prediction of Transcription Factor-Target Gene Binding Sites

The promoters of the genes involved in muscle growth were obtained from the *Siniperca chuatsi* genome (http://genomes.igb-berlin.de/cgi-bin/hgGateway?db=sinChu7 (accessed on 21 May 2024)), and the IDs for each gene are presented in Table 2. Then, Jaspar was used to predict the binding site of *Twist2* to the promoter of the target gene (*Twist2* motif ID: MA0633.3), and the relative profile score threshold was set at 90%.

## 3. Results

### 3.1. Fast Muscle Fiber Changes During Tissue Repair After Chinese Perch Muscle Injury

**Figure 1:**
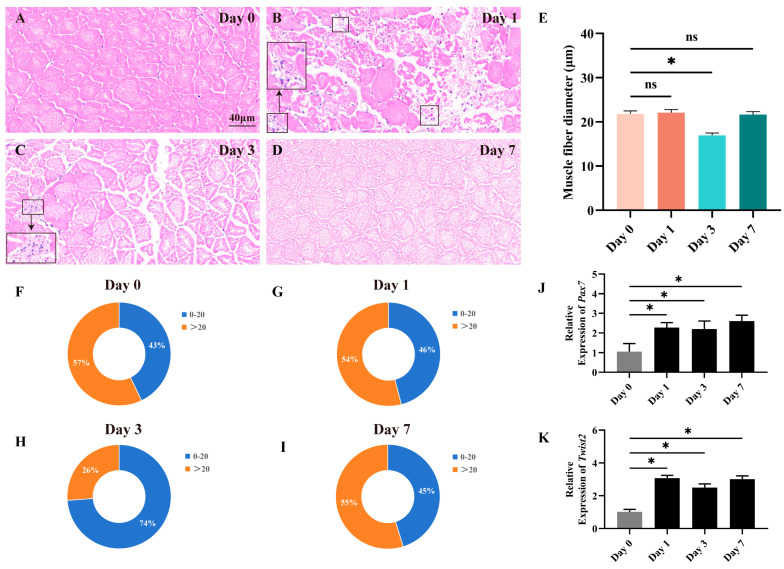
Histological section analysis of the fast muscle during the damage and repair process. HE staining shows cross-sections of fast muscle on days 0, 1, 3, and 7 of muscle injury (**A**–**D**). Inflammatory cells have been marked with black square frames (**B**,**C**). Cross-sectional area distribution of muscle fibers on days 0, 1, 3, and 7 after muscle injury (**E**). Frequency distribution of muscle fiber diameters on days 0, 1, 3, and 7 after muscle injury (**F**–**I**). The expression of *Pax7* and *Twist2* after muscle injury in Chinese perch (**J**,**K**). Values in graphs are mean ± SE, *n* = 3. * Indicates a significant difference between groups (*p* < 0.05). ns stands for no significant difference (*p* > 0.05).

In the constructed muscle injury model, fast muscle tissue repair was assessed by histological methods on days 0, 1, 3, and 7. The results showed that injection of CTX rapidly caused muscle injury. In the progression of fast muscle injury, distinct morphological changes were observed (Figure 1A–D). On the 1st day, muscle fiber structures became disordered, with portions of the tissue fragmented. Concurrently, inflammatory cells infiltrated, marking the onset of early-stage injury. By the 3rd day, new muscle fibers began to emerge. As the injury advanced to the 7th day, muscle fibers regained regular alignment, nearing normalcy. The newly formed muscle fibers matured, leading to the restoration of muscle morphology and structure. Through the analysis of the distribution of myofiber cross-sectional area and diameter, we observed an increase in the proportion of myofibers with a diameter less than 20 μm on day 3, indicating significant changes in myofiber size following the experimental manipulation (Figure 1E–I). The findings indicate that muscle damage in Chinese perch can lead to alterations in myofiber morphology and size, which are subsequently repaired through the hyperplasia of new myofibers. The expression of *Pax7* and *Twist2* genes during the regeneration and repair of fast muscle tissue was assessed using RT-qPCR. The results revealed a significant upregulation of both *Pax7* and *Twist2* expression on the first day following CTX injection, with their high expression levels maintained thereafter (Figure 1J–K). The findings revealed that the expression pattern of *Twist2* closely mirrored that of *Pax7* throughout the process of fast muscle tissue regeneration and repair.

### 3.2. Effect of Inhibition of Twist2 on Gene Expression Related to Muscle Growth

**Figure 2:**
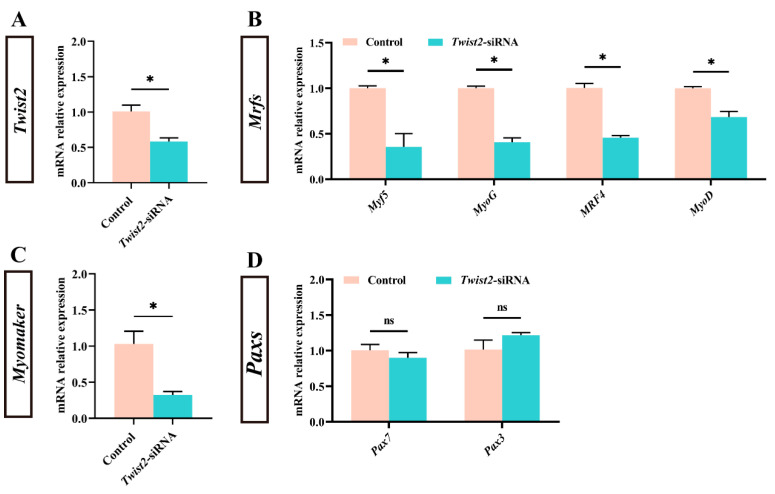
Effect of *Twist2*-siRNA injection on the expression of genes related to muscle growth. The relative expression of *Twist2* (**A**). The relative expression of *Myf5*, *MyoG*, *MRF4* and *MyoD* (**B**). The relative expression of *Myomaker* (**C**). The relative expression of *Pax7* and *Pax3* (**D**). Values are plotted as mean ± SE, *n* = 6. * Indicates the significant difference between the control and the *Twist2*-siRNA groups (*p* < 0.05). ns stands for no significant difference (*p* > 0.05).

To investigate the effect of *Twist2* on the expression of genes related to muscle growth of Chinese perch, the expression of myogenic regulator factors and *Pax* family genes in muscle was analyzed following the interference with the *Twist2* gene. The expression level of *Twist2* was significantly reduced in the muscle of the *Twist2*-siRNA group (*p* < 0.05) (Figure 2A). In addition, the expression levels of the myogenic regulatory factors *MyoD*, *MyoG*, *MRF4,* and *Myf5* also showed a significant decrease in the muscle of Chinese perch, while the expression levels of *Pax3* and *Pax7* were not significantly changed (Figure 2B–D). The results suggested that interference with *Twist2* affected the expression of myogenic regulator factors in Chinese perch muscle.

### 3.3. Histological Analysis of Fast Muscle After Inhibition of Twist2

**Figure 3:**
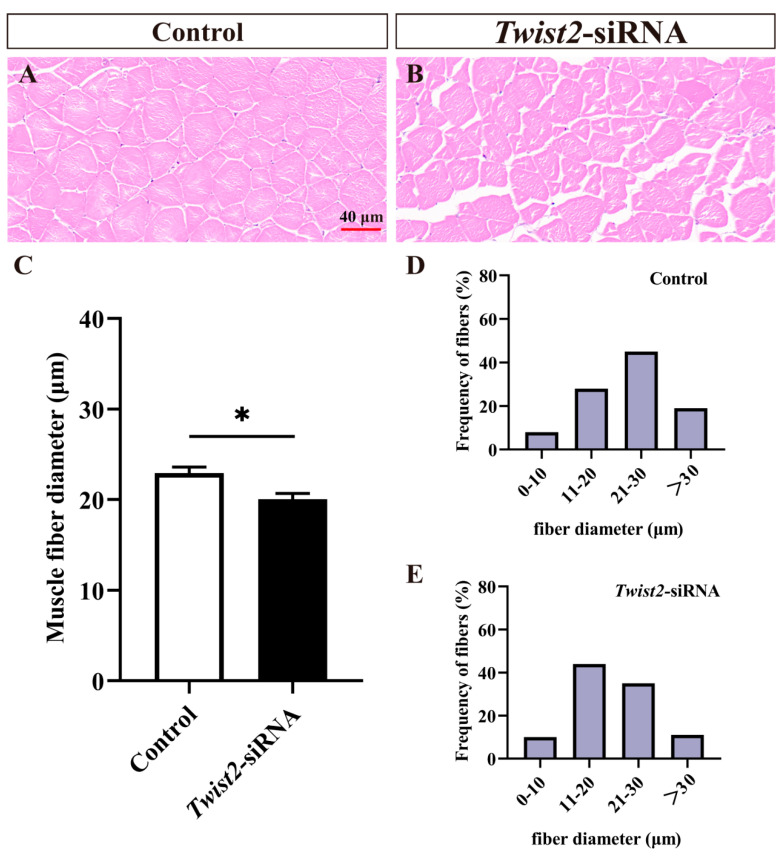
Histological section analysis of fast muscle in the control and *Twist2*-siRNA groups. HE staining showed cross-sections of fast muscles in the control and *Twist2*-siRNA groups (**A**,**B**). The muscle fiber diameters in the control and *Twist2*-siRNA groups (**C**). Frequency distribution of muscle fiber diameters in the control and *Twist2*-siRNA groups (**D**,**E**). Values in the figures are the mean ± SEM, *n* = 6. * Indicates the significant difference in expression between the control and *Twist2*-siRNA groups (*p* < 0.05).

Fast muscle section analysis showed that muscle fiber diameters were significantly reduced in the *Twist2*-siRNA group in comparison with the control group (Figure 3A–C). The muscle fiber diameters in the control group were mainly distributed in 20–30 µm (Figure 3D), while those of muscle fibers in the *Twist2*-siRNA group were mainly distributed in 10–20 µm (Figure 3E). The results indicated that *Twist2* played an important role in the process of muscle fiber thickening in Chinese perch.

### 3.4. Effects of Interfering with Twist2 on the Proliferation of Myoblasts

**Figure 4:**
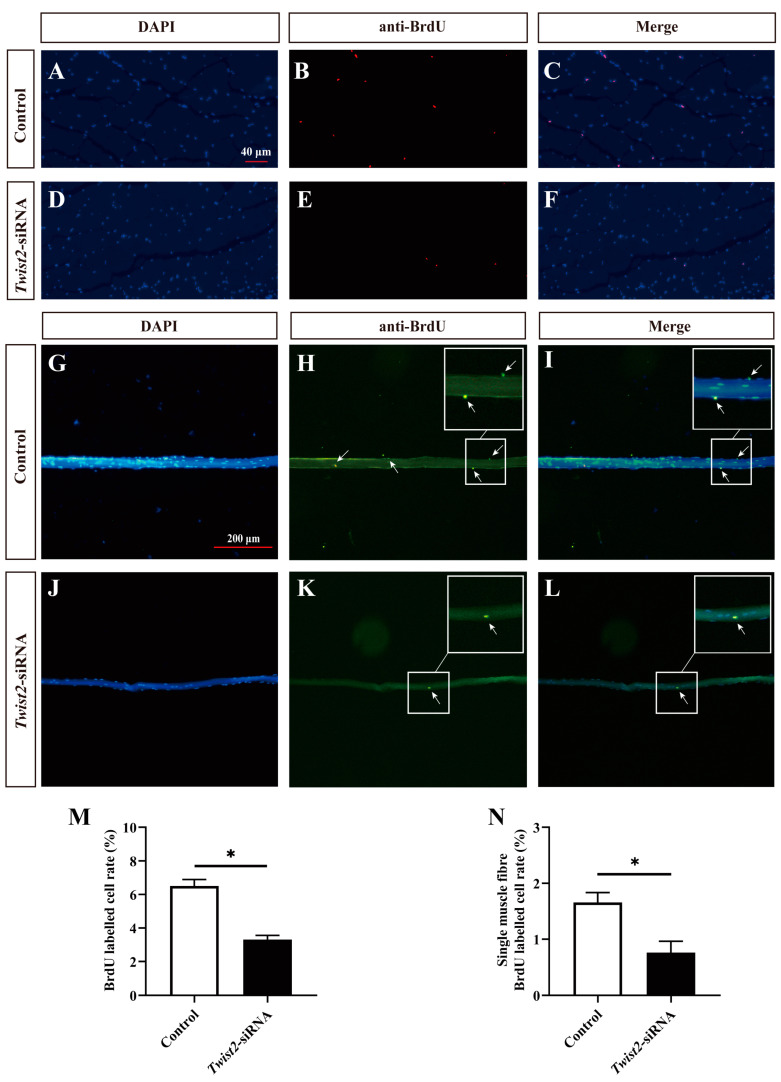
Effects of interfering with *Twist2* expression on myoblast proliferation. Immunofluorescence labeling of the transverse section of muscle fibers in the control and *Twist2*-siRNA groups (**A**–**F**). Immunofluorescent labeling of single muscle fibers in the control and *Twist2*-siRNA groups (**G**–**L**). Nuclei were labeled with DAPI (blue), proliferating cells in cross-sections of muscle fibers were labeled with BrdU antibody (red), and proliferating cells of single muscle fibers were labeled with BrdU antibody (green), the arrows indicate BrdU positive cells. (**M**) Comparison of proliferating cell frequencies in muscle fiber cross-sections between the control and *Twist2*-siRNA groups. (**N**) Comparison of proliferating cell frequencies of single muscle fibers in the control and *Twist2*-siRNA groups. The scale bars for muscle fiber cross-sections and single muscle fibers were 40 µm and 200 µm, respectively. * Indicates the significant difference in expression between the control and *Twist2*-siRNA groups (*p* < 0.05).

The proliferation of myoblasts was evaluated by immunofluorescence labeling on the cross-section of muscle fibers and single muscle fibers. BrdU-stained positive cells were regarded as proliferating cells. The findings indicated that the number of proliferating myoblasts was significantly decreased in the *Twist2*-siRNA group after inhibition of *Twist2* expression compared with the control group (Figure 4). The results indicated that *Twist2* plays an important role in the proliferation of myoblasts in Chinese perch.

### 3.5. The Analysis of Nuclei Number in Single Muscle Fibers

**Figure 5:**
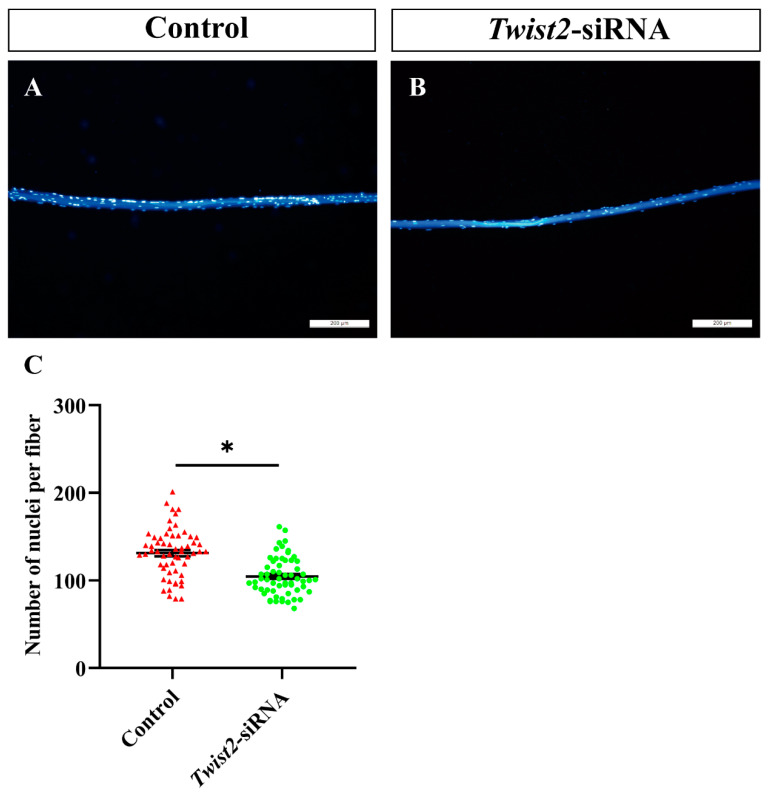
Analysis of the number of nuclei in single myofiber cells in the control and *Twist2*-siRNA groups. Single muscle fibers from the control (**A**) and *Twist2*-siRNA (**B**) groups were isolated and stained with DAPI (blue). Statistical analysis of nuclear numbers in muscle fibers from the control and *Twist2*-siRNA groups (**C**). Values in the figures are the mean ± SEM, *n* = 60. Each red triangle and each green circle represent the number of individual nuclei in the control and *Twist2*-siRNA groups, respectively. * Indicates the significant difference between the control and the *Twist2*-siRNA groups (*p* < 0.05).

To further explore the role of *Twist2* in myoblast fusion, the nuclei on single muscle fibers were stained with DAPI. The findings indicated a significant decrease in the number of nuclei on single muscle fibers in the *Twist2*-siRNA group compared with the control group (Figure 5). This study demonstrated that the inhibition of *Twist2* expression impedes myoblast fusion, consequently resulting in a decrease in myofiber diameter.

### 3.6. Prediction of Twist2 Interactions with Target Gene Promoters

**Figure 6:**
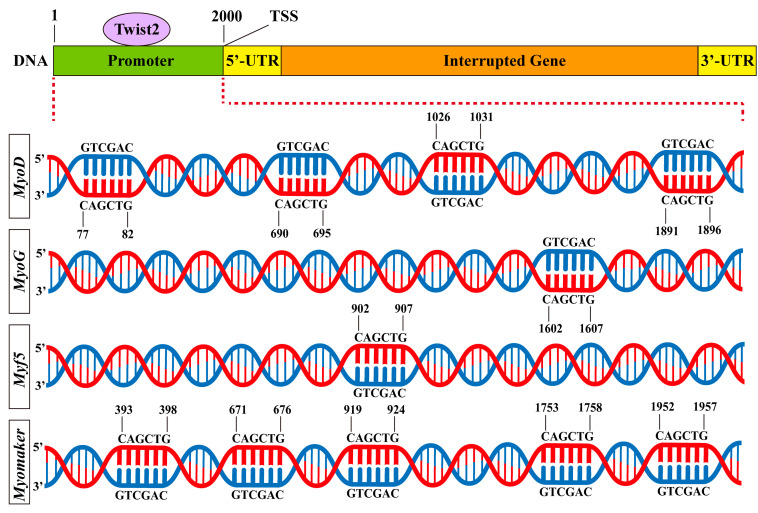
Predicted binding sites for muscle growth-related genes to Twist2.

To examine the interaction of the transcription factor Twist2 with various muscle growth-related target genes, Jaspar was used to predict the binding of Twist2 to the promoter sites of each target gene. The results showed that Twist2 had different numbers of binding sites with *MyoD*, *Myf5, MyoG,* and *Myomaker* genes (Figure 6). This suggests that Twist2 may regulate the transcription of *MyoD*, *Myf5, MyoG,* and *Myomaker* genes.

## 4. Discussion

Investigating the regulatory mechanisms behind skeletal muscle growth is crucial for offering essential scientific and technological foundations, as well as theoretical insights, which are vital for genetically enhancing economic animal breeds, improving muscle quality, and boosting overall production efficiency [27,28]. Fish, as one of the economically important animals, exhibit a distinct trajectory of skeletal muscle growth compared to mammals and avians [29]. Unlike mammals and avian, where postnatal muscle growth relies solely on the hypertrophy of pre-existing muscle fibers [30,31,32], numerous fish species experience a combination of myofiber hyperplasia and hypertrophy for muscle growth after hatching [33]. Currently, muscle growth-related studies are mainly focused on model animals such as mice (*Mus musculus*) and zebrafish (*Danio rerio*); however, the molecular regulatory mechanisms of muscle growth in economic fish have not been fully identified [34,35,36,37]. Focusing on economically valuable fish species as the primary subject of cultivation and enhancing their muscle growth can lead to accelerated growth rates and improved productivity in aquaculture. In this study, we found that inhibition of *Twist2* leads to a decrease in the proliferation and fusion of myoblasts in the fast muscle of juvenile Chinese perch, which suggests that *Twist2* is crucial for promoting the proliferation and fusion of myoblasts during the post-embryonic stage of the Chinese perch.

It has been demonstrated that a variety of transcription factors are involved in the regulation of myogenesis, for example, myogenic regulatory factors (MRFs) and myogenic transcription factors (Pax3, Pax7) [38,39,40,41]. Additionally, multiple studies have revealed the role of *Twist2* in the regulation of muscle growth [42,43,44]. Previous studies have shown that satellite cells located beneath the muscle basal lamina and expressing *Pax7* are considered to be the only muscle-resident cell population with innate myogenic potential [45]. However, Liu et al. in 2017 challenged this view by describing a second population of myogenic progenitor cells in a mouse study that are located within the myofiber mesenchyme and express the transcription factor Twist2 [21]. They found that cells expressing *Twist2* specialize in the repair and maintenance of type IIx/b muscle fibers in vivo, and the Twist2^+^ progenitor cells are highly myogenic in vitro, fusing with both their own and satellite cells [21]. In the present study, we assessed the effect of the regenerative repair phase of muscle-damaged tissues in Chinese perch and found that the proportion of newly proliferated myoblasts increased and the regenerative capacity of muscle fibers was enhanced at 24 h after CTX injection. In addition, the expression pattern of *Twist2* showed a high degree of consistency with that of *Pax7* in this study. Pax7, a signature gene of satellite cells, stimulates the differentiation of satellite cells to the muscle lineage during muscle regeneration [46]. Although Twist2 labeled a whole new class of myogenic progenitor cells molecularly and anatomically distinct from satellite cells, Twist2^+^ cells were found to be equally capable of autonomously initiating myogenesis during regeneration [21]. In the mouse model, during muscle regeneration, the population of Twist2^+^ cells initially decreased promptly; however, the count of Twist2^+^ cells rebounded swiftly by day 7 following CTX treatment [21]. The above results suggest that in mice, Twist2-positive cells can be seen to play a role in the regenerative repair process, with a reduced number in the repair phase, possibly differentiating into other cells, such as myoblasts, which are then involved in the fusion. In our study, *Twist2* and *Pax7* expression showed a significant up-regulation on the first day after CTX administration and remained at a high level up to seven days. Based on the above experimental results, it suggests that *Twist2* may be involved in the process of muscle tissue regeneration and repair in Chinese perch. In this process, *Twist2* may play a similar biological function to that of *Pax7*. The specific mechanism of the role of *Twist2* in the muscle growth of Chinese perch after hatching still needs to be verified by further experiments.

In mice, lineage tracing using tamoxifen-induced insertion of the CreERT2 transgene into the *Twist2* locus revealed that Twist2^+^ mesenchymal stromal cells are able to fuse into myofibers, and ablation of Twist2^+^ cells throughout the body results in significant atrophy of type IIb fibers [21]. This result suggests that Twist2^+^ mesenchymal cells are important for the maintenance of type IIb fibers. In this study, in order to investigate the role of *Twist2* in fast muscle in juvenile Chinese perch, we employed *Twist2*-siRNA to interfere with the expression of the *Twist2* gene in the fast muscle of juvenile Chinese perch. The results showed that the expression of myogenic regulatory factor family genes (*MyoD*, *MyoG*, *MRF4*, and *Myf5*) and muscle fusion *Myomaker*, which are associated with the regulation of muscle growth, were also significantly down-regulated compared to the control group, except for *Pax3* and *Pax7*. This suggests that interfering with *Twist2* during the juvenile stage of Chinese perch affects the normal expression pattern of fast muscle growth-regulated genes. MRFs are able to drive the expression of muscle genes in the process of myogenesis and are considered to be major transcription factors up-regulated during myogenesis, which can affect the differentiation of stem cells into myogenic lineage cells [38,47]. Moreover, inhibition of *Twist2* leads to a decrease in the number of proliferating myoblasts. Therefore, the inhibition of *Twist2* might prevent the differentiation of myogenic stem cells and inhibit the proliferation of myoblasts. Whether the new class of *Twist2*-labeled myogenic progenitor cells differentiated into myoblasts requires further validation. *Myomaker* was reported to directly regulate myoblast fusion and muscle formation [23,48]. Predictions of the interaction of *Twist2* with the promoters of the target genes indicate that *Twist2* can directly regulate *MyoD*, *MyoG*, *Myf5,* and *Myomaker*. The results suggest that Twist2 may directly regulate the expression of *MyoD*, *MyoG*, *Myf5,* and *Myomaker* and affect the proliferation, differentiation, and fusion of myoblasts, thereby contributing to the growth of fast muscle fibers in the post-hatching stage of Chinese perch.

We have preliminarily investigated the functional role of *Twist2* in the fast muscle growth of Chinese perch during the post-embryonic stage. Compared with common model animals such as mice and Drosophila, we found that *Twist2* has both similarities and differences in its regulatory role in certain processes of muscle growth. For example, in mice, in addition to the aforementioned functions of *Twist2* in muscle growth, its expression can maintain myogenic progenitor cells in an undifferentiated state, preparing for the initiation of muscle growth [21]. In Drosophila, *Twist2* promotes myogenesis, activates muscle development-related genes, and drives mesodermal cells to differentiate into muscle cells [49]. We speculate that the differences in the role of *Twist2* in muscle growth among different organisms may be due to variations in growth patterns and species. Moreover, the regulation of *Twist2* on cell growth signaling pathways forms a complex network, with its regulatory targets including pathways such as HB-EGF-EGFR, Notch, and AKT/GSK-3β, which collectively influence cell growth [50,51,52]. In summary, our findings provide a theoretical basis for the efficient aquaculture of Chinese perch. Further research will be conducted on the regulatory mechanisms of *Twist2* in Chinese perch to support the sustainable development of the aquaculture industry.

## 5. Conclusions

Overall, our findings suggest that Twist2 plays a crucial role in stimulating the proliferation and fusion of myoblasts and promoting the hypertrophy of myofibers during the juvenile phase of Chinese perch. This research may offer novel insights into uncovering the molecular mechanisms underlying muscle growth in fish and assist in the cultivation of new fish species with superior growth traits.

## Figures and Tables

**Table 1:** The primers for RT-qPCR.

Genes	Forward Primer Sequence (5′-3′)	Reverse Primer Sequence (5′-3′)	Product Size (bp)	TM Value (°C)
*Twist2*	AAGCTCGCCTCCAGATACAT	GCTGACATAGACCAAGCACC	152	58.6
*MyoD*	CAACGACGCCTTTGAGACCCTG	GTCCGAATCCCGCTGTAGTGT	177	61.7
*MyoG*	CGAGACCAACCCTTACTTCTTCCCT	GACTCCCACACAAGCCCATCAT	137	61.3
*MRF4*	AGACCAACCCTTATCTTTCAATG	CGGTCTCGGACGGAACATTAT	153	57.8
*Myf5*	AGGTCAACCACGCTTTCGAG	GTTTTCCACCTGCTCCCGTA	140	58.5
*Pax3*	TGAACCCCGCCATAGGAAAC	TCAGAGGGGAGATGGCGTAG	105	59
*Pax7*	AGCCACAACATGACTTCTCC	GTCCACCGTCTTAATGGAGG	119	55
*Myomaker*	AGTGTTTACGGCACGGCTC	CGTGGTCAACACTCCAAACAT	105	57.6
*Rpl13*	CACAAGAAGGAGAAGGCTCGGGT	TTTGGCTCTCTTGGCACGGAT	120	62.1

**Table 2:** Target gene promoter information.

Genes	Gene ID	Promoter Length
*MyoD*	SC7-LG05_06085	2 kb
*MyoG*	SC7-LG04_05388	2 kb
*Myf5*	SC7-LG15_19245	2 kb
*Myomaker*	SC7-LG07_09935	2 kb

## Data Availability

All data generated or analyzed during this study are included in this published article. Additional data related to this study are available from the corresponding author upon reasonable request.
